# Complete genome sequence and transcriptomic analysis of a novel marine strain *Bacillus weihaiensis* reveals the mechanism of brown algae degradation

**DOI:** 10.1038/srep38248

**Published:** 2016-11-30

**Authors:** Yueming Zhu, Peng Chen, Yunjuan Bao, Yan Men, Yan Zeng, Jiangang Yang, Jibin Sun, Yuanxia Sun

**Affiliations:** 1 National Engineering Laboratory for Industrial Enzymes, Tianjin Institute of Industrial Biotechnology, Chinese Academy of Sciences, Tianjin 300308, China

## Abstract

A novel marine strain representing efficient degradation ability toward brown algae was isolated, identified, and assigned to *Bacillus weihaiensis* Alg07. The alga-associated marine bacteria promote the nutrient cycle and perform important functions in the marine ecosystem. The *de novo* sequencing of the *B. weihaiensis* Alg07 genome was carried out. Results of gene annotation and carbohydrate-active enzyme analysis showed that the strain harbored enzymes that can completely degrade alginate and laminarin, which are the specific polysaccharides of brown algae. We also found genes for the utilization of mannitol, the major storage monosaccharide in the cell of brown algae. To understand the process of brown algae decomposition by *B. weihaiensis* Alg07, RNA-seq transcriptome analysis and qRT-PCR were performed. The genes involved in alginate metabolism were all up-regulated in the initial stage of kelp degradation, suggesting that the strain Alg07 first degrades alginate to destruct the cell wall so that the laminarin and mannitol are released and subsequently decomposed. The key genes involved in alginate and laminarin degradation were expressed in *Escherichia coli* and characterized. Overall, the model of brown algae degradation by the marine strain Alg07 was established, and novel alginate lyases and laminarinase were discovered.

Marine environments are not only formidable in natural conditions, but are rich in diverse polysaccharides and organic matter. Bacterial organisms inhabiting these environments have adapted to these hostile habitats during their long history of evolution. Ultimately, evolutionary adaptation confers the bacteria with the capability to degrade marine-specific biomass, optimize energy metabolism, and synthesize natural products.

Algae are widely distributed in marine environments and contain large amounts of polysaccharides, many of which are unique to seaweeds. For instance, brown algae contain carboxylated polysaccharide (alginate)^1^, anionic sulfated polysaccharide (fucoidan)^2^, and laminarin^3^, all of which are not found in land plants. Hence, numerous bacterial species isolated from seaweeds exhibit alga-associated lifestyle by enzymatic catabolism of algal constituents. The end products of polysaccharide degradation can be further stored as energy or used for the biosynthesis of other macromolecules of high economic value. For efficient utilization of carbohydrates and other nutrients, marine bacteria also evolved to form specialized transporter systems for the uptake of various nutrient molecules pertaining to marine niches. Nutrient molecules are imported into the cytoplasm or periplasm for further metabolism by specific enzymes in a regulated manner. Owing to the high content of polysaccharides in brown algae, engineered microbial platforms have also been constructed to produce biofuel using brown macroalgae^4^,^5^,^6^.

Alginate is a major component of brown algae. The content of alginate varies with species and seasons, and the highest content reaches to 50% in dry cell weight^7^. Alginate is a high-molecular-weight polymer composed of two types of β-1,4-linked monosaccharides, such as β-d-mannuronic acids and α-l-guluronic acids. The chemical linkage can form contiguous polymers of β-d-mannuronic acids (PolyM), α-l-guluronic acids (polyG), or random heteropolymers of the two monosaccharides (PolyMG). Alginate degradation is usually initiated by lytic depolymerization via β-elimination reaction involving alginate lyases^8^. The lytic products, oligoalginate, are further degraded into unsaturated monosaccharides (spontaneously rearranged into 4-deoxy-l-erythro-5-hexoseulose uronic acid, DEH) by exo-type lyases. DEH is converted into 2-keto-3-deoxy-6-phosphogluconate (KDPG) via hydrogenation and phosphorylation reaction, which is catalyzed by DEH reductase and 2-keto-3-deoxy-gluconate (KDG) kinase respectively. KDPG is cleaved into pyruvate and d-glyceraldehyde 3-phosphate by KDPG aldolase and utilized through the Entner-Doudoroff pathway. The complete alginate metabolic pathway in marine bacterium has been described^9^.

Laminarin and mannitol are the storage saccharides of brown algae. Laminarin is a linear polysaccharide made up of β-1,3-glucan with occasional β-1,6-linked branches. Hence, laminarin can be depolymerized by β-1,3-glucanases and debranching enzymes. Various β-1,3-glucanases that can degrade laminarin have been found in bacteria, archaea, and eukaryotes^10^,^11^,^12^,^13^, and some of these glucanases are called laminarinase. The final products of hydrolysis mainly include glucose, laminaribiose, and laminaritriose^14^. The approaches of mannitol absorption and utilization by microbes are relatively clear^15^.

Recently, we isolated a novel *Bacillus* species Alg07 from the marine area of Weihai City, Shandong Province, China^16^. This strain shows extremely high extracellular activity toward alginate and extraordinary metabolic ability to degrade brown algae. Notably, phylogenetic analysis based on 16 S rRNA gene sequences suggests that Alg07 may be a novel species within the *Bacillus* genus. To elucidate the metabolic capability and ecological adaptation of Alg07 with respect to brown algae degradation, we sequenced the complete genome of *Bacillus weihaiensis* Alg07 and performed gene annotation and carbohydrate-active enzyme (CAZyme) analysis. Furthermore, RNA-seq transcriptomic analysis and qRT-PCR were employed to determine the expression levels of key genes in brown algae degradation. The activities of key genes were also confirmed by heterologous expression and activity assay.

## Materials and Methods

### Bacterial growth condition

LB medium was used to cultivate *B. weihaiensis* Alg07 for genome extraction and incubate *E. coli* for the expression of key genes. Carbon source utilization tests were carried out using a medium containing 10 g/l tryptone, 5 g/l beef extract, 3 g/l NaCl, 2 g/l Na_2_HPO_4_·12H_2_O, and 2.4 mg/l bromothymol blue with 10 g/l monosaccharide (l-arabinose, d-xylose, d-glucose, d-fructose, d-galactose, d-mannose, and d-mannitol), disaccharide (maltose, sucrose, trehalose, lactose, and cellobiose), or polysaccharide (starch, cellulose, laminarin, agar, and agarose). To determine the differential gene expression profile of this strain with brown seaweed, Alg07 was cultivated in the modified Marine Broth 2216 medium containing 5 g/l tryptone, 1 g/l yeast extract, 5 g/l (NH_4_)_2_SO_4_, 19.45 g/l NaCl, 12.6 g/l MgCl_2_·6H_2_O, 6.64 g/l MgSO_4_·7H_2_O, 0.55 g/l KCl, 0.16 g/l NaHCO_3_, and 0.1 g/l ferric citrate with 10 g/l kelp powder, as well as in the medium without kelp powder as the control. All chemicals and reagents used in this study were of analytical grade.

### Genome sequencing and gene annotation

The genome of *B. weihaiensis* Alg07 was purified using a Bacterial DNA Kit (Omega) and sequenced on the PacBio RS II platform (Pacific Biosciences, Menlo Park, CA) with a post-filter mean read length of 9.3 kbp. Sequencing was performed at Wuhan Institute of Biotechnology. De novo assembly was performed using the Hierarchical Genome Assembly Process 2.2.0 (HGAP 2.2.0)^17^ workflow, and the complete circular genome was derived. Gene prediction and functional annotation were carried out via RAST (Rapid Annotation using Subsystem Technology). Genome annotation was performed by comparison against public databases NCBI NR^18^, SwissProt^19^, COG^20^, SEED^21^, KEGG^22^, and CAZy databases^23^.

### Phylogenetic analysis

The 16 S rRNA sequences from *Bacillus* species were downloaded from the Silva ribosomal RNA database. Only the entries with similarity >93% were used for further analysis. Multiple sequence alignment of 16 S rRNA was performed using ClustalW. To reduce the influence of incomplete 16 S rRNA sequences from databases, the first 27 and the last 36 alignment loci were removed. The phylogenetic tree was constructed using Neighbor-Joining method and implemented in the Molecular Evolutionary Genetics Analysis (MEGA) software version 6.0. Bootstrap support values were obtained by generating 1000 replicate trees.

### Transcriptomic analysis

The total RNA of *B. weihaiensis* Alg07 was purified using an RNAprep pure Kit for cell/bacteria (Tiangen). RNA integrity was assessed using the RNA Nano 6000 Assay Kit of the Agilent Bioanalyzer 2100 system (Agilent Technologies). Sequencing libraries were generated using NEBNext Ultra Directional RNA Library Prep Kit for Illumina (NEB), and sequenced on an Illumina Hiseq 2500 platform. Sequencing was performed at Beijing Novogene Bioinformatics Technology Co., Ltd. The raw sequence data were filtered by removing reads containing adapter, reads containing poly-N, and low-quality reads. The clean reads were aligned with the genome of *B. weihaiensis* Alg07 by using Bowtie2–2.2.3. The sequencing and alignment of each sample were carried out in triplicate. Gene expression was quantified as reads per kilobase of coding sequence per million reads (RPKM) algorithm. Genes with an adjusted P-value < 0.05 found by DESeq were assigned as differentially expressed.

### qRT-PCR analysis

To confirm the RNA-Seq results and explain the process of brown algae degradation, the expression change of 19 genes were determined by qRT-PCR using the primers listed in Table S1. First-strand cDNA was synthesized from total RNA extracted after 12, 24, and 48 h incubation using HiScript II 1st Strand cDNA Sythesis Kit (Vazyme). cDNA was used as template for qRT-PCR with ChamQ SYBR qPCR Master Mix (Vazyme). The 16 s rRNA gene was used as an endogenous control. All reactions were carried out in triplicate.

### Activity assay of key genes

Key genes involved in alginate and laminarin degradation were amplified using the primers listed in Table S1 and cloned into the expression vector pET-21a(+). The recombinant plasmids were transformed into *E.coli* BL21(DE3) cells. The recombinant *E. coli* cells were cultured in LB medium. After being induced by IPTG, cells were harvested and disrupted by sonication. Supernatants were used as crude extract for activity assays. The activities of *E. coli* BL21 with the empty vector pET-21a were used as control.

To determine the activities of alginate lyase and laminarinase, 200 μl of appropriately diluted enzyme was added into 1.8 ml of Tris-HCl buffer (20 mM, pH 8.0) containing 10 g/l sodium alginate or laminarin. After incubation at 40 °C for 20 min, the samples were heated in boiling water for 5 min and then cooled in cold water. The supernatant was analyzed by 3,5-dinitrosalicylic acid (DNS) method^24^.

Crude enzyme was added to Tris-HCl buffer (20 mM, pH 8.0) containing 1 g/l DEH and 1 mM NADPH to determine the activities of DEH reductase. Reaction was initiated by the addition of enzyme to the substrate solution, and reaction progress was monitored by measuring the decrease in absorbance at 340 nm caused by the oxidation of NADPH to NADP^+^.

### Statistical analysis

For the RNA-seq study, differential expression analyses of two conditions were performed using the DESeq R package (1.18.0). DESeq provides statistical routines for determining differential expression in digital gene expression data by using a model based on the negative binomial distribution. The resulting P-values were adjusted using Benjamini and Hochberg’s approach for controlling the false discovery rate. Genes with an adjusted P-value < 0.05 found by DESeq were assigned as differentially expressed. For the qRT-PCR study, a P-value < 0.05 was considered statistically significant.

### Nucleotide sequence accession number

The genome sequence of *B. weihaiensis* Alg07 has been submitted to the GenBank (accession No. CP016020 and CP016021). The RNA-seq data have been deposited in the NCBI Short Read Archive (SRA) database (accession No. SRP076196).

## Results

### Phylogenetic analysis of the marine strain Alg07

**Figure 1:**
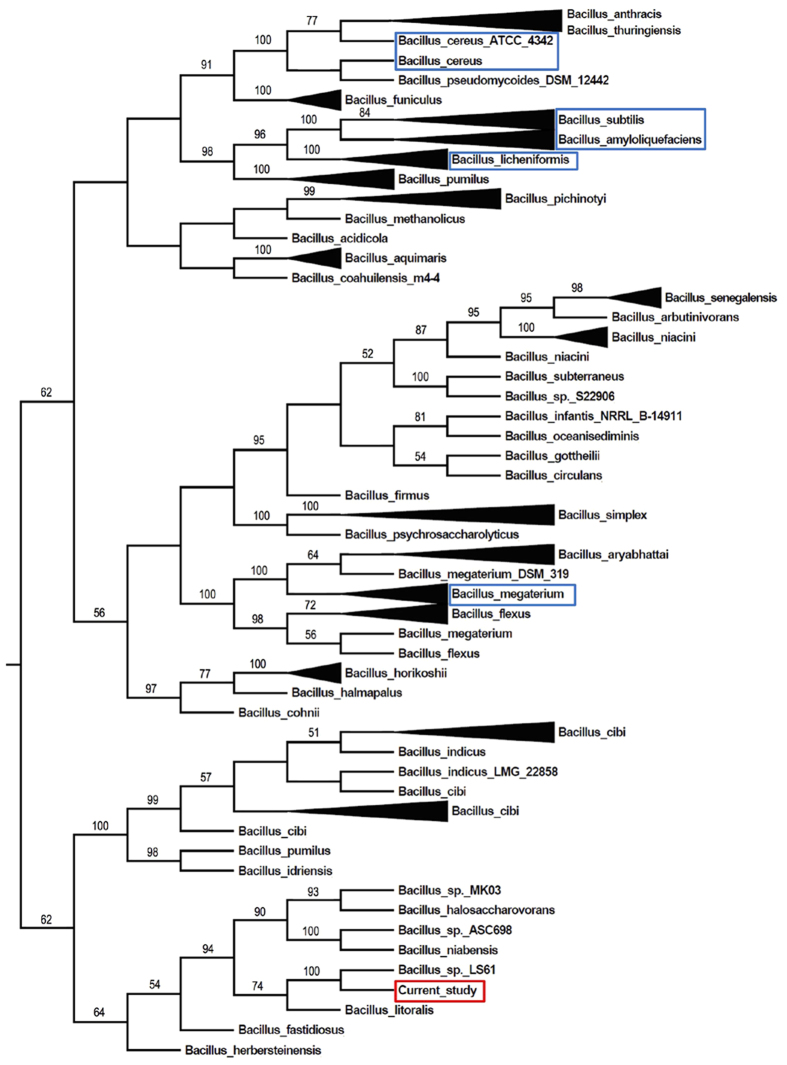
Phylogenetic tree of 16 S rRNA sequences from *B. weihaiensis* Alg07 and other known *Bacillus* species. The trees containing the same species were collapsed and indicated with triangles. Several well-studied *Bacillus* species are highlighted in blue frame.

Phylogenetic analysis based on 16 S rRNA gene sequences showed that the Alg07 strain falls within the radiation cluster of *Bacillus* species but forms a distinct lineage (Fig. 1). The Alg07 strain is the most closely related to *Bacillus litoralis*, with similarities of 96–97%. Therefore, Alg07 may represent a strain type of a novel species within the genus *Bacillus*, and the name *B. weihaiensis* was assigned. Alg07 exhibits lower similarity (<95%) with several other well-studied *Bacillus* species, such as *Bacillus subtilis, Bacillus cereus, Bacillus licheniformis*, and *Bacillus megaterium*, indicating its distinct genotypic properties when compared with these species.

### Genomic properties of *B. weihaiensis* Alg07

**Figure 2:**
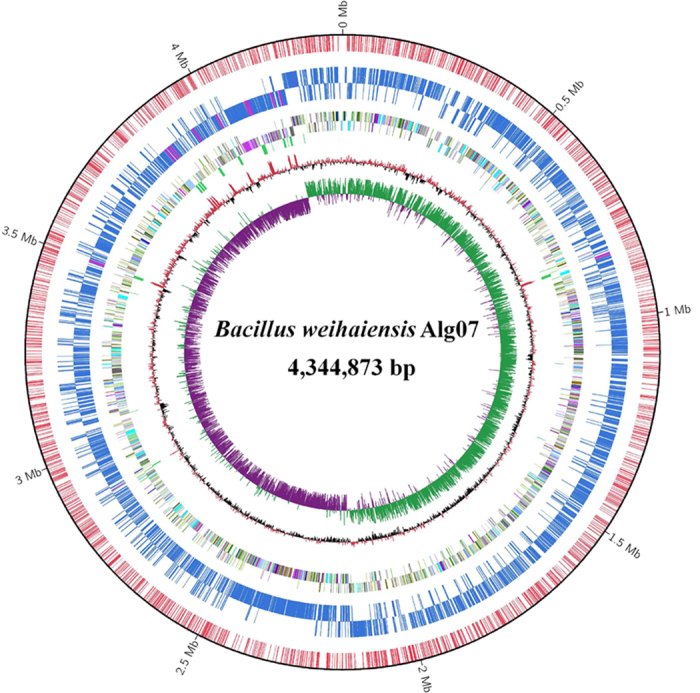
Circular representation of the genome map of *B. weihaiensis* Alg07. The circles from the innermost to the outermost: Circle 1 for GC skew (G−C)/(G+C); Circle 2 for GC content; Circle 3 for rRNA and tRNA; Circles 4 and 5 for protein coding genes in the reverse and forward strands respectively; Circles 6 and 7 for N4-methylcytosine (m4C) and N6-methyladenosine (m6A) sites in CDS/rRNA/tRNA in the reverse and forward strands respectively; and Circle 8 for m4C and m6A sites in intergene regions.

The genome of *B. weihaiensis* Alg07 was sequenced on the PacBio RS II platform. Through the HGAP method, the PacBio RS II data were de novo assembled to two contigs with 106.75 × depth of coverage. The large contig is the chromosome of *B. weihaiensis* Alg07, which contains 4,344,873 bp with an average G+C content of 36.48% (Fig. 2). The small contig containing 16,403 bp forms an end-to-end overlap structure, which suggests that a plasmid exists in the cell of Alg07. The plasmid could be extracted using a plasmid extraction kit (Fig. S1). The genome encodes 4267 predicted open reading frames (ORFs), of which 1061 were annotated with unknown functions. A total of 11 rRNA operons and 105 tRNAs were found in the genome.

### Mono- and disaccharide utilization

Based on the result of gene annotation, *B. weihaiensis* Alg07 might utilize various mono- and disaccharides, such as d-ribose, d-xylose, d-glucose, d-fructose, d-galactose, d-mannose, d-mannitol, maltose, lactose, and trehalose. Carbohydrate uptake mainly occurs through the ATP-binding cassette (ABC) transport system or the phosphotransferase system (PTS). The genes encoding catabolic enzymes and corresponding transporters are organized in the loci and listed in Table S2. However, the enzymes involved in the utilization of l-arabinose, l-rhamnose, d-sorbose, and d-sorbitol are not found in the genome of *B. weihaiensis* Alg07. These annotation results were corroborated by the carbon source utilization tests with selected mono- and disaccharides. Mannitol, which is the major monosaccharide in brown algae cells, might be converted to fructose-6-phosphate by mannitol-1-phosphate 5-dehydrogenase (MPDH, gene 423) and funneled into the EMP pathway.

### Identification of polysaccharide utilization loci using the CAZy website

According to the results of carbon source utilization tests, the Alg07 strain could degrade algal polysaccharides, such as agarose, alginate, starch, and laminarin. To determine the key enzymes involved in polysaccharide degradation, diverse CAZymes in the *B. weihaiensis* Alg07 genome were identified, including 32 glycoside hydrolases (GHs), 31 glycosyl transferases (GTs), 3 polysaccharide lyases (PLs), 29 carbohydrate esterases (CEs) and 23 carbohydrate-binding modules (CBMs) (Table S3). As reported, alginate lyase, which is responsible for alginate degradation, belongs to the PL family^25^. Three polysaccharide lyases were found in the genome of Alg07 (Table 1), and two enzymes belonging to the PL15 family were annotated as oligoalginate lyases (Bw806 and Bw2030). The protein encoded by Bw1998 was annotated as an F5/8 type C domain protein and belonged to the PL17 family. Further domain detection based on PFAM database^26^ showed that this protein contains six domains: one heparinase II/III-like domain, two calcium-activated chloride channel domains, one fibronectin type III domain, one F5/8 type C domain, and one Gram-positive anchor domain. Moreover, based on the signal peptide prediction with SignalP v4.1^27^, the potential alginate lyase is a secretory protein, whereas the potential oligoalginate lyases do not have any signal peptides. We suggest that the alginate lyase is secreted into the extracellular environment and cleaves alginate into oligoalginate which is then transported to the cell and further degraded by oligoalginate lyase.

Furthermore, α/β-amylase, β-glucanase, pullulanase, and β-glucosidase were found in the genome of *B. weihaiensis* Alg07 (Table 1). These enzymes belong to the GH family and may be involved in the hydrolysis of glucans (e.g. starch), laminarin, and cellulose in brown algae. The endo-β-1,3–1,4 glucanase (Bw2859) and β-glucanase (Bw3263 and Bw3268) might be potential laminarinases that can break down the β-1,3-glucosidic linkages, while pullulanases (Bw919 and Bw2732) might be responsible for removing β-1,6-linked branches. We also found that the β-glucosidase (Bw3267) and β-glucanase precursor (Bw3268) together with an *o*-Glycosyl hydrolase family 30 protein (Bw3265) and a hypothetical protein (Bw3266) were organized in a gene locus.

### Transcriptomic analysis of *B. weihaiensis* Alg07 in kelp-containing culture

*B. weihaiensis* Alg07 was cultivated in the modified Marine Broth 2216 medium with 10 g/l kelp powder, and the alginate lyase activities of the culture supernatant were determined at different time intervals. The maximum activity was observed at 20 h after inoculation. Considering that mRNAs are rapidly degraded after protein translation, we extracted the total RNA of the Alg07 cells after incubation for 12 h and used RNA-seq transcriptomic analysis to examine the important genes for brown algae degradation. RNA-seq analysis indicates that 104 genes displayed statistically significant mRNA level changes (adjusted P value < 0.05); 34 of the genes displayed increased transcript levels (Table S4), and 70 displayed decreased transcript levels (Fig. S2).

By comparing the expression values between the *B. weihaiensis* Alg07 cultivated with and without kelp, we were able to identify which genes might be involved in kelp degradation. The expression level of Bw1998 was significantly increased, suggesting that the enzyme might be an alginate lyase. The expression levels of genes in one cluster (from Bw800 to Bw807) were significantly increased, except for Bw804. This gene cluster encodes an ABC-type polysaccharide transport system composed of a permease (Bw800), an integral membrane protein (Bw801), and a substrate-binding protein (Bw802), which could transport oligoalginates into the cell. The transcription of genes for the ABC transport system might be activated by a two-component system constituted by a response regulator (Bw803) and a sensor kinase (Bw804). The expression level of the potential oligoalginate lyase (Bw806) was increased, but that of Bw2030 was not changed. Bw806 is expected to be a functional oligoalginate lyase, which could cleave oligoalginate to generate DEH. Bw805, annotated as 3-oxoacyl-(acyl-carrier-protein) reductase, might act as a DEH reductase, which converts DEH into KDG. KDG could be further phosphorylated and decomposed by KDG kinase (Bw503) and KDPG aldolase (BW504), whose expression level increased. The expression of key genes involved in alginate assimilation and degradation was significantly induced at 12 h (Table 2), but those of key genes involved in laminarin and mannitol degradation showed no changes. This result may be explained as *B. weihaiensis* Alg07 decomposing only alginate to disrupt the cell wall in the initial stage of brown algae degradation.

### qRT-PCR analysis

**Figure 3:**
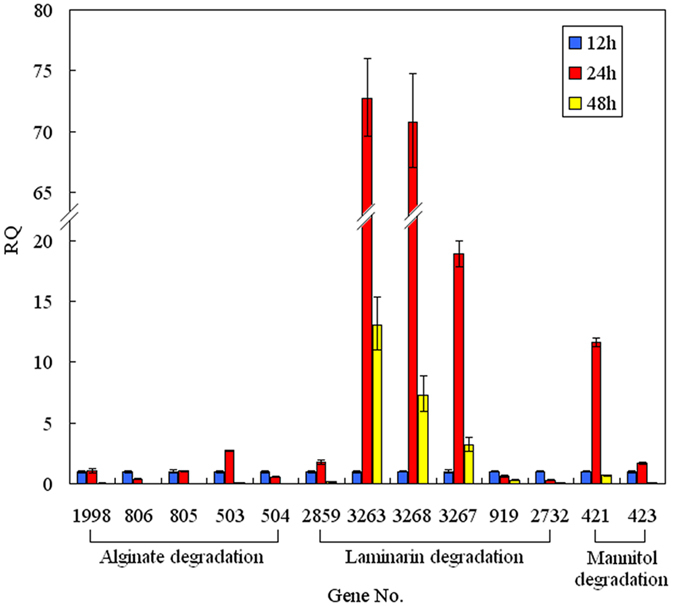
Expression levels of key genes in Alg07 cells after 12, 24 and 48 h incubation identified by qRT-PCR analysis. The 12 h incubation sample was set as the reference (RQ value was 1). RQ: Relative Quantification.

Nine genes involved in alginate degradation and displaying increased transcript levels according to RNA-seq analysis were verified by qRT-PCR. The results were in good agreement with those from the RNA-seq analysis (Table 2). To further discover induced genes involved in laminarin and mannitol degradation, qRT-PCR was carried out using total RNA extracted from Alg07 cells after 12, 24, and 48 h of incubation. The sample incubated for 12 h was set as the reference. Results indicate that the expression level of genes involved in laminarin and mannitol degradation is significantly increased in the 24 h incubation sample (Fig. 3). For laminarin degradation, three genes were highly induced. Bw3263 and Bw3268 function in degrading laminarin into oligo-laminarin, whereas the function of Bw3267 is degrading oligo-laminarin into glucose. However, the Bw2732 and Bw919, which were annotated as pullulanase, were expressed at a lower level, suggesting that these enzymes might not perform an important function in the degradation of branched laminarin. For mannitol degradation, the significantly increased gene was Bw421, which was annotated as mannitol-specific PTS system. Bw423, which was annotated as MPDH, was slightly increased. In the 48 h incubation sample, the expression levels of all genes except for Bw3263, Bw3268, and Bw3267 were significantly decreased. This finding suggests that the laminarin is not completely degraded at 48 h, perhaps because of the high content of laminarin or the low activities of these enzymes.

### Heterologous expression and activity assay of key genes

The key genes involved in the degradation of alginate and laminarin were cloned and expressed in *E.coli* (Fig. S3). Cell extracts were used as crude enzymes to determine the activities of the recombinant proteins. Bw1998 exhibited extremly high alginate lyase activity, and the properties of the alginate lyase were characterized in detail (Fig. S4). Alginate lyase could depolymerize both polyM and polyG and completely degrade alginate into oligoalginate. The novel alginate lyase is a high-molecular-weight protein with multiple domains. Thus, we constructed the truncated protein containing only the heparinase II/III-like domain and compared its activity with the full-length protein. The results indicate that the truncated and full-length proteins are not significantly different, suggesting that the activity is independent of noncatalytic modules (data not shown).

We also found that Bw806 can degrade oligoalginate to monosaccharides, whereas Bw2030 cannot. These results are consistent with the transcriptomic analysis. Bw805 showed high activity toward purified DEH and NADPH. The percentages of amino acid sequence identity between the novel DEH reductase and those reported from *Flavobacterium* sp. UMI-01^28^ and *Sphingomonas* sp. A1^29^,^30^ are 40.08% and 33.08% respectively. The catalytic tetrad Asn-Tyr-Lys-Ser and the cofactor-binding sequence motif Thr-Gly-X-X-X-Gly-X-Gly were also found in Bw805 (Fig. S5). The activities of potential laminarinases (Bw2859, Bw3263, and Bw3268) were determined by DNS method with laminarin as the substrate. All enzymes showed activity toward laminarin, with that of Bw3268 being the highest.

## Discussion

The Alg07 strain was isolated from rotting seaweed on the coast of Weihai City. The alginolytic community was enriched using modified Marine Broth 2216 containing sodium alginate, and the single colonies presenting high activity toward alginate were selected and identified. Further study found that the Alg07 strain can degrade kelp pieces into sludge and show good growth in the medium with kelp as the energy source. This finding indicates that this marine bacterium has evolved versatile abilities in degrading brown algae and adapted to the nutritional condition of the marine environment. Alga-associated marine bacteria perform important functions in the protection and preservation of marine ecosystems^31^,^32^. These bacteria can decompose algae that may not be degraded by marine animals and convert seaweed components into storage energy (e.g. glycogen and the genes involved in glycogen synthesis of Alg07 are listed in Table S5) that might provide nutrients for the marine animals. Hence, the carbon cycle could be promoted by this kind of marine bacteria. Marine bacteria are also rich sources of various important biocatalysts, such as alga-specific polysaccharidases, esterases, proteases, dehalogenases, and so on^33^,^34^.

**Figure 4:**
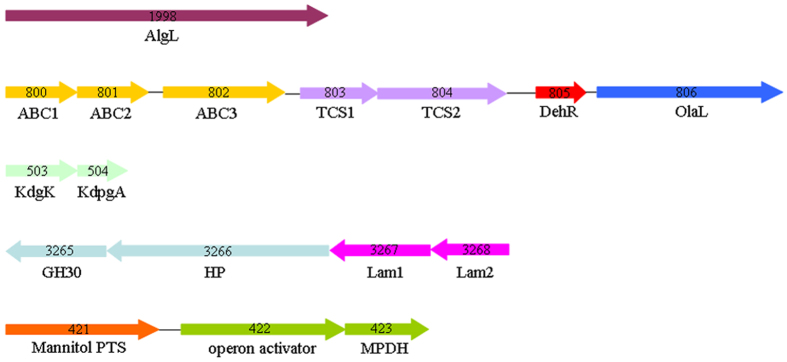
Loci for alginate, laminarin, and mannitol utilization of *B. weihaiensis* Alg07. AlgL, alginate lyase; ABC, ABC transporter components; TCS, two-component system; DehR, DEH reductase; OlaL, oligoalginate lyase; KdgK, KDG kinase; KdpgA, KDPG aldolase; HP, hypothetical protein; Lam1, β-glucosidase; Lam2, laminarinase; mannitol PTS, mannitol specific PTS system; MPDH, mannitol-1-phosphate 5-dehydrogenase.

**Figure 5:**
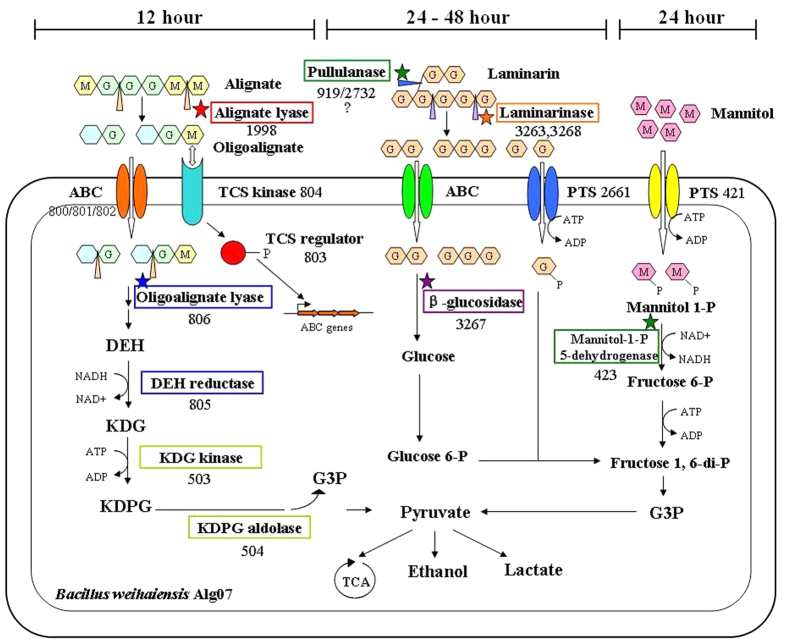
Model of brown algae degradation by *B. weihaiensis* Alg07.

According to phylogenetic analysis, the Alg07 strain is a novel *Bacillus* species. Given that the strain was isolated in Weihai City, the strain was named as *Bacillus weihaiensis* Alg07. Genome sequencing and transcriptome analysis of the strain were conducted to study the degradation mechanism of brown algae and discover novel enzymes from this marine microbe. We found novel enzymes and gene clusters (Fig. 4) involved in the degradation of algal polysaccharides and established the degradation model of brown algae by *B. weihaiensis* Alg07 (Fig. 5). First, the Alg07 strain was triggered to synthesize and secrete alginate lyase (Bw1998) when cultured with brown algae (e.g. kelp). Alginate lyase degrades alginate into oligoalginate, which might activate the two-component system. As reported, the phosphorylated response regulator was predicted to promote the expression of the neighboring ABC transport system^35^,^36^. Consequently, the oligoalginate was transported into the cells and further degraded by oligoalginate lyase and DEH reductase. Together with those of the two-component system, genes that are responsible for oligosaccharide transport and metabolism (from oligoalginate to KDG) were located in gene cluster II. The genes encoding KDG kinase and KDPG aldolase were located in the cluster III. The two enzymes are important for the complete degradation of alginate.

Alginate degradation might lead to the destruction of the cell wall of brown algae, causing laminarin and mannitol to leak out easily. The degradation processes of laminarin and mannitol are relatively simple compared to those of alginate. Gene clusters IV and V show the genes involved in the degradation of laminarin and mannitol respectively (Fig. 4). Laminarinase and pullulanase could cleave laminarin into mono-, di-, and trisaccharides. Monosaccharide, namely, glucose, is taken up by the glucose-specific PTS system and metabolized by the EMP pathway. Di- and trisaccharides were further degraded to glucose by intracellular β-glucosidase. Mannitol is transported by the mannitol-specific PTS system and simultaneously phosphorylated to form mannitol 1-phosphate, which is subsequently dehydrogenated by MPDH and enters the EMP pathway.

**Figure 6:**
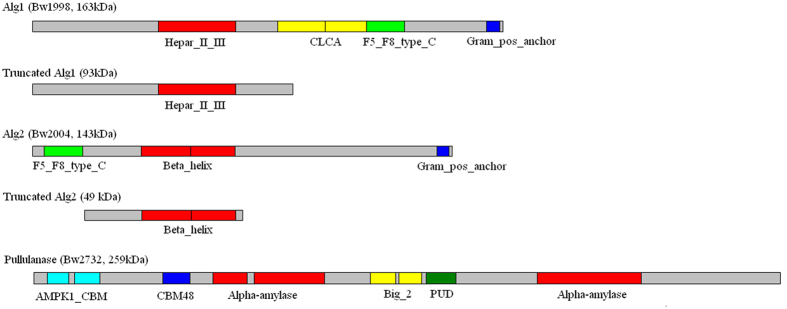
Domain architectures of the high-molecular-weight enzymes in *B. weihaiensis* Alg07. Hepar_II_III, heparinase II/III-like protein; CLAL, calcium-activated chloride channel; F5_F8_type_C, F5/8 type C domain; Gram_pos_anchor, Gram-positive anchor; Beta_helix, right-handed beta helix region; AMPK1_CBM, glycogen recognition site of AMP-activated protein kinase; CBM48, carbohydrate-binding module 48; Alpha-amylase, α-amylase catalytic domain; Big_2, bacterial Ig-like domain (group 2); PUD, bacterial pullanase-associated domain.

Alginate depolymerization is the initial stage for the efficient utilization of the brown algae, therefore, the function of alginate lyase is crucial. Various alginate lyases from marine bacteria have been discovered^37^,^38^,^39^,^40^, and several of these lyases have been structurally characterized^41^,^42^,^43^. Most endolytic bacterial alginate lyases are assigned to families PL-5 and PL-7, whereas exolytic oligoalginate lyases are grouped into PL-15 and PL-17 families. Although the alginate lyase (Bw1998) from *B. weihaiensis* Alg07 belongs to the PL-17 family, Bw1998 could cleave alginate into oligosaccharides. Moreover, the sequence alignment results indicate that this protein shares extremely low identity with other reported alginate lyases. Interestingly, the molecular weight of this enzyme is very high (163 kDa), and multiple domains are found in this protein. The activity and substrate preference between the full-length and truncated Bw1998 showed no difference, which confirmed that the activity is due to the heparinase II/III-like domain. In other words, the alginate lyase is a part of the full-length protein. We speculate that this protein is the precursor of the alginate lyase and that the mature enzyme is formed through post-translational modifications. Other multi-domain proteins were likewise found in the genome of strain Alg07, for example, Bw2004 and Bw2732 (Fig. 6). Bw2004 was annotated as a polyguluronate lyase precursor and could not be classified to any PL family. However, the precursor showed activity toward alginate and preferred PolyM to PolyG. We also constructed the truncated protein, which only contained the beta helix region, and compared its activity with the full-length protein. The results are similar with those of Bw1998 (data not shown). Hence, two novel alginate lyases were found in the genome of the Alg07 strain. However, the results of transcriptome analysis and qRT-PCR indicate that the expression of Bw2004 was not induced when the strain Alg07 was cultured in the medium containing kelp. Bw1998 appears to be preferentially produced by the strain because the enzyme showed no preference toward polyM and ployG and could degrade alginate more effectively.

In conclusion, we found the key enzymes and pathways involved in algal polysaccharide degradation in *B. weihaiensis* Alg07 and explained the degradation process of brown algae by the marine bacterium. High-molecular-weight enzymes in the strain were extraordinary and interesting. The post-translational modification and structures of the novel alginate lyases will be characterized in our further study.

## Additional Information

**How to cite this article**: Zhu, Y. *et al*. Complete genome sequence and transcriptomic analysis of a novel marine strain *Bacillus weihaiensis* reveals the mechanism of brown algae degradation. *Sci. Rep.* **6**, 38248; doi: 10.1038/srep38248 (2016).

**Publisher’s note:** Springer Nature remains neutral with regard to jurisdictional claims in published maps and institutional affiliations.

## Supplementary Material

Supplementary Information

## Figures and Tables

**Table 1:** Potential enzymes for algal polysaccharide degradation.

Gene ID	CAZy family	Annotation	Substrate
1998	PL17	F5/8 type C domain protein	alginate
806	PL15	oligo alginate lyase	oligo alginate
2030	PL15	oligo alginate lyase	oligo alginate
88	GH14	Beta-amylase	(1–4)-alpha-D-glucosidic linkages to terminal non-reducing residues, starch
3188	GH13	Periplasmic alpha-amylase	(1–4)-alpha-D-glucosidic linkages, starch
2859	GH16	Endo-beta-1,3-1,4 glucanase (Licheninase)	(1–3)- or (1–4)-beta-D-glucosidic linkages, laminarin, lichenin
3263	GH16	Beta-glucanase precursor	(1–3)-beta-D-glucosidic linkages, laminarin
3268	GH16	Beta-glucanase precursor	(1–3)-beta-D-glucosidic linkages, laminarin
919	GH13	Glycogen debranching enzyme/Pullulanase	(1–6)-alpha-D-glucosidic linkages, laminarin
2732	GH13	Pullulanase	(1–6)-alpha-D-glucosidic linkages, laminarin
3267	GH1	Beta-glucosidase	beta-D-glucosides to terminal non-reducing residues, laminarin, cellulose

**Table 2:** Induced genes involved in alginate degradation determined by RNA-Seq and qRT-PCR.

Gene ID	log_2_FoldChange (by RNA-Seq)	2^−ΔΔct^ (by qRT-PCR)	Annotation	Predicted Function
1998	4.19	3.38	alginate lyase	To degrade alginate into oligoalginate
800	6.20	5.92	ABC-type polysaccharide transport system, permease component	To transport oligoalginate into cells
801	6.93	14.04	putative transport system integral membrane protein	
802	4.24	25.82	ABC transporter, substrate-binding protein	
803	—	—	two-component response regulator yesN	To activate ABC transport system
804	3.30	2.48	two-component system sensor kinase	
805	5.99	21.69	DEH reductase	To transform DEH to KDG
806	6.23	52.08	oligo alginate lyase	To degrade oligoalginate into DEH
807	5.97	8.62	pectin degradation protein	unknown
503	5.27	1.48	2-keto-3-deoxy-gluconate kinase	KDG metabolism
504	6.16	7.32	2-keto-3-deoxy-phosphogluconate aldolase	

## References

[b1] Lee K. Y. & Mooney D. J. Alginate: properties and biomedical applications. Prog Polym Sci 37, 106–126 (2012). 22125349 10.1016/j.progpolymsci.2011.06.003 PMC3223967

[b2] Li B., Lu F., Wei X. & Zhao R. Fucoidan: structure and bioactivity. Molecules 13, 1671–1695 (2008). 18794778 10.3390/molecules13081671 PMC6245444

[b3] Kadam S. U., Tiwari B. K. & O′Donnell C. P. Extraction, structure and biofunctional activities of laminarin from brown algae. Int J Food Sci Tech 50, 24–31 (2015).

[b4] Takeda H., Yoneyama F., Kawai S., Hashimoto W. & Murata K. Bioethanol production from marine biomass alginate by metabolically engineered bacteria. Energy Environ Sci 4, 2575–2581 (2011).

[b5] Wargacki A. J. . An engineered microbial platform for direct biofuel production from brown macroalgae. Science 335, 308–313 (2012). 22267807 10.1126/science.1214547

[b6] Enquist-Newman M. . Efficient ethanol production from brown macroalgae sugars by a synthetic yeast platform. Nature 505, 239–243 (2014). 24291791 10.1038/nature12771

[b7] Kraan S. Algal polysaccharides, novel applications and outlook. Carbohydrates-Comprehensive studies on glycobiology and glycotechnology. Biochemistry, Genetics and Molecular Biology Carbohydrates. InTech Press, pp 489–532 (2012).

[b8] Wong T. Y., Preston L. A. & Schiller N. L. ALGINATE LYASE: review of major sources and enzyme characteristics, structure-function analysis, biological roles, and applications. Annu Rev Microbiol 54, 289–340 (2000). 11018131 10.1146/annurev.micro.54.1.289

[b9] Kim H. S., Lee C. G. & Lee E. Y. Alginate Lyase: Structure, Property, and Application. Biotech Biopro Eng 16, 843–851 (2011).

[b10] Alderkamp A. C., van Rijssel M. & Bolhuis H. Characterization of marine bacteria and the activity of their enzyme systems involved in degradation of the algal storage glucan laminarin. FEMS Microbiol Ecol 59, 108–117 (2007). 17233748 10.1111/j.1574-6941.2006.00219.x

[b11] Gueguen Y., Voorhorst W. G., van der Oost J. & de Vos W. M. Molecular and biochemical characterization of an endo-beta-1,3-glucanase of the hyperthermophilic archaeon *Pyrococcus furiosus*. J Biol Chem 272, 31258–31264 (1997). 9395451 10.1074/jbc.272.50.31258

[b12] Giese E. C. . Enzymatic hydrolysis of botryosphaeran and laminarin by β-1,3-glucanases produced by *Botryosphaeria rhodina* and *Trichoderma harzianum* Rifai. Process Biochem 41, 1265–1271 (2006).

[b13] Lépagnol-Descamps V. . Purification and determination of the action pattern of Haliotis tuberculata laminarinase. Carbohydr Res 310, 283–289 (1998). 9821264 10.1016/s0008-6215(98)00181-5

[b14] Krah M. . The laminarinase from thermophilic eubacterium *Rhodothermus marinus* – conformation, stability, and identification of active site carboxylic residues by site-directed mutagenesis. Eur J Biochem 257, 101–111 (1998). 9799108 10.1046/j.1432-1327.1998.2570101.x

[b15] Wisselink H. W. . Overproduction of heterologous mannitol 1-phosphatase: a key factor for engineering mannitol production by *Lactococcus lactis*. Appl Environ Microbiol 71, 1507–1514 (2005). 15746354 10.1128/AEM.71.3.1507-1514.2005 PMC1065179

[b16] Chen P. . Screening and identification of a bacterial strain and optimization of medium composition and culture conditions for the production of alginate lyase. Food Science 36, 105–111 (2015).

[b17] Chin C. S. . Nonhybrid, finished microbial genome assemblies from long-read SMRT sequencing data. Nat Methods 10, 563–569 (2013). 23644548 10.1038/nmeth.2474

[b18] Pruitt K. D., Tatusova T., Brown G. R. & Maglott D. R. NCBI Reference Sequences (RefSeq): current status, new features and genome annotation policy. Nucleic Acids Res 40, D130–D135 (2012). 22121212 10.1093/nar/gkr1079 PMC3245008

[b19] Magrane M. & Consortium U. UniProt Knowledgebase: a hub of integrated protein data. Database. bar009. (2011). 21447597 10.1093/database/bar009 PMC3070428

[b20] Tatusov R. L., Galperin M. Y., Natale D. A. & Koonin E. V. The COG database: a tool for genome-scale analysis of protein functions and evolution. Nucleic Acids Res 28, 33–36 (2000). 10592175 10.1093/nar/28.1.33 PMC102395

[b21] Henry C. S. . High-throughput generation, optimization and analysis of genome-scale metabolic models. Nat Biotech 28, 977–982 (2010). 10.1038/nbt.1672 20802497

[b22] Kanehisa M., Goto S., Kawashima S., Okuno Y. & Hattori M. The KEGG resource for deciphering the genome. Nucleic Acids Res 32, D277–D280 (2004). 14681412 10.1093/nar/gkh063 PMC308797

[b23] Park B. H., Karpinets T. V., Syed M. H., Leuze M. R. & Uberbacher E. C. CAZymes Analysis Toolkit (CAT): web service for searching and analyzing carbohydrate-active enzymes in a newly sequenced organism using CAZy database. Glycobiology 20, 1574–1584 (2010). 20696711 10.1093/glycob/cwq106

[b24] Miller G. L. Use of dinitrosalicylic acid reagent for determination of reducing sugar. Anal Chem 31, 426–428 (1959).

[b25] Zhu B. & Yin H. Alginate lyase: Review of major sources and classification, properties, structure-function analysis and applications. Bioengineered 6, 125–131 (2015). 25831216 10.1080/21655979.2015.1030543 PMC4601208

[b26] Sonnhammer E. L., Eddy S. R. & Durbin R. Pfam: a comprehensive database of protein domain families based on seed alignments. Proteins 28, 405–420 (1997). 9223186 10.1002/(sici)1097-0134(199707)28:3<405::aid-prot10>3.0.co;2-l

[b27] Petersen T. N., Brunak S., von Heijne G. & Nielsen H. SignalP 4.0: discriminating signal peptides from transmembrane regions. Nat Methods 8, 785–786 (2011). 21959131 10.1038/nmeth.1701

[b28] Inoue A., Nishiyama R., Mochizuki S. & Ojima T. Identification of a 4-deoxy-L-erythro-5-hexoseulose uronic acid reductase, FlRed, in an alginolytic bacterium *Flavobacterium* sp. strain UMI-01. Mar Drugs 13, 493–508 (2015). 25603344 10.3390/md13010493 PMC4306948

[b29] Takase R., Ochiai A., Mikami B., Hashimoto W. & Murata K. Molecular identification of unsaturated uronate reductase prerequisite for alginate metabolism in *Sphingomonas* sp. A1. Biochim Biophys Acta 1804, 1925–1936 (2010). 20685299 10.1016/j.bbapap.2010.05.010

[b30] Takase R., Mikami B., Kawai S., Murata K. & Hashimoto W. Structure-based conversion of the coenzyme requirement of a short-chain dehydrogenase/reductase involved in bacterial alginate metabolism. J Biol Chem 289, 33198–33214 (2014). 25288804 10.1074/jbc.M114.585661 PMC4246080

[b31] Azam F. & Malfatti F. Microbial structuring of marine ecosystems. Nat Rev Microbiol 5, 782–791 (2007). 17853906 10.1038/nrmicro1747

[b32] Ramanan R., Kim B. H., Cho D. H., Oh H. M. & Kim H. S. Algae-bacteria interactions: Evolution, ecology and emerging applications. Biotechnol Adv 34, 14–29 (2016). 26657897 10.1016/j.biotechadv.2015.12.003

[b33] Martin M., Portetelle D., Michel G. & Vandenbol M. Microorganisms living on macroalgae: diversity, interactions, and biotechnological applications. Appl Microbiol Biotechnol 98, 2917–2935 (2014). 24562178 10.1007/s00253-014-5557-2

[b34] De Santi C., Altermark B., de Pascale D. & Willassen N. P. Bioprospecting around Arctic islands: Marine bacteria as rich source of biocatalysts. J Basic Microbiol 56, 238–253 (2016). 26662844 10.1002/jobm.201500505

[b35] Joseph P., Fichant G., Quentin Y. & Denizot F. Regulatory relationship of two-component and ABC transport systems and clustering of their genes in the *Bacillus/Clostridium* group, suggest a functional link between them. J Mol Microbiol Biotechnol 4, 503–513 (2002). 12432961

[b36] Shulami S. . A two-component system regulates the expression of an ABC transporter for xylo-oligosaccharides in *Geobacillus stearothermophilus*. Appl Environ Microbiol 73, 874–884 (2007). 17142383 10.1128/AEM.02367-06 PMC1800775

[b37] Yoon H. J. . Overexpression in *Escherichia coli*, purification, and characterization of *Sphingomonas* sp. A1 alginate lyases. Protein Expr Purif 19, 84–90 (2000). 10833394 10.1006/prep.2000.1226

[b38] Matsushima R. . Analysis of extracellular alginate lyase and its gene from a marine bacterial strain, *Pseudoalteromonas atlantica* AR06. Appl Microbiol Biotechnol 86, 567–576 (2010). 19844705 10.1007/s00253-009-2278-z

[b39] Inoue A. . Characterization of an alginate lyase, FlAlyA, from *Flavobacterium* sp. strain UMI-01 and its expression in *Escherichia coli*. Mar Drugs 12, 4693–4712 (2014). 25153766 10.3390/md12084693 PMC4145338

[b40] Han W. . Novel alginate lyase (Aly5) from a polysaccharide-degrading marine bacterium, *Flammeovirga* sp. strain MY04: effects of module truncation on biochemical characteristics, alginate degradation patterns, and oligosaccharide-yielding properties. Appl Environ Microbiol 82, 364–374 (2015). 26519393 10.1128/AEM.03022-15 PMC4702622

[b41] Yamasaki M. . Structure and function of a hypothetical *Pseudomonas aeruginosa* protein PA1167 classified into family PL-7: a novel alginate lyase with a beta-sandwich fold. J Biol Chem 279, 31863–31872 (2004). 15136569 10.1074/jbc.M402466200

[b42] Ogura K., Yamasaki M., Mikami B., Hashimoto W. & Murata K. Substrate recognition by family 7 alginate lyase from *Sphingomonas* sp. A1. J Mol Biol 380, 373–385 (2008). 18514736 10.1016/j.jmb.2008.05.008

[b43] Dong S. . Molecular insight into the role of the N-terminal extension in the maturation, substrate recognition, and catalysis of a bacterial alginate lyase from polysaccharide lyase family 18. J Biol Chem 289, 29558–29569 (2014). 25210041 10.1074/jbc.M114.584573 PMC4207973

