# Supplementary material for: Complete genome sequence and transcriptomic analysis of a novel marine strain Bacillus weihaiensis reveals the mechanism of brown algae degradation
Source: Sci Rep. 2016 Nov 30;6:38248. doi: 10.1038/srep38248 (PMC5128808; doi:10.1038/srep38248)

**Complete genome sequence and transcriptomic analysis of a novel marine strain *Bacillus* *weihaiensis* reveals the mechanism of brown algae degradation**

Yueming Zhu^1^, Peng Chen^1^, Yunjuan Bao, Yan Men, Yan Zeng, Jiangang Yang, Jibin Sun, Yuanxia Sun^*^

National Engineering Laboratory for Industrial Enzymes, Tianjin Institute of Industrial Biotechnology, Chinese Academy of Sciences, Tianjin 300308, China

^*^Corresponding Author:

Yuanxia Sun, telephone: +86-22-84861960, fax: +86-22-84861961, e-mail: [syx0430@hotmail.com](mailto:syx0430@hotmail.com)

^1^The first two authors contributed equally to this paper.

**Supplementary information**

**Table S1. Primers used in this study**

| **Name** | **Sequence (5’ to 3’)** | **Usage** |
| --- | --- | --- |
| 16sF | GCGGTAATACGTAGGTGGCA | qRT-PCR |
| 16sR | TTTCCAATGACCCTCCACGG |
| 800F | GCAAAGTGTTGGTTGGGGAA |
| 800R | AGCGATTTGCTCCATCCACT |
| 801F | TGGGCTTTAGTTATCCCGTCT |
| 801R | CGCGCTGCGTCAATTAAAGT |
| 802F | TTCCTGAAGAAGCGTGGGAG |
| 802R | CGAATCCAAGTAGCACGACG |
| 803F | TGGAATCGAAGCAGACGAACT |
| 803R | ATGTTGGTCAGCCCATTTGC |
| 804F | AACGGCAAAGGGATGTCACC |
| 804R | ACATTCGCGATACCAATCCC |
| 805F | CGTTACGTCGGCAGCATTTA |
| 805R | CATCATGTGCTGCAAGAGAGC |
| 806F | TTGGTGGGACTTCCCATTCG |
| 806R | GTGCTACCCAACCAACGTCT |
| 807F | AATTGGGAACAAGCAGAGCCA |
| 807R | TGTGCATACCCTTCCGCTC |
| 1998F | ACATCCTGATGCCAAGTGGG |
| 1998R | AGCTGGCGCCTGTGTTATTA |
| 503F | TGTTGCAGTTGGTCTGGCTA |
| 503R | TTTCCAAACTCGTCAGCTCCT |
| 504F | TGCTTTTGAAAACGGAGCGG |
| 504R | TGGAATCTGCGGAAGTGGAC |
| 2859F | GAGCCTTCACAACCCAATGC |
| 2859R | CCCAAATCTCGTGGTCAAACG |
| 3263F | TGGCTGTAGGAGGCTGGTAT |
| 3263R | TCGAGCTCATATACGCGGAC |
| 3268F | TTCACGGTAGAATGGGAGCC |
| 3268R | CCAGCCTTTGTTCGCTCACT |
| 3267F | TAAAACGGGAATGGGGTGGG |
| 3267R | AGGCTGCACCATTTTCGGTA |
| 421F | TGCTTTCATAGCGTGGGGTT |
| 421R | ACGTTACCATCGGTCCAACT |
| 423F | TGAGTGGGTTGTGGAAACGA |
| 423R | TGCATGGCCTGTATTAACCGT |
| 919F | TCCTTTTCTACACGCTGGGC |
| 919R | AACAGTTCTGGCTTGCCACT |
| 2732F | ACGCGATTGTTCCAGGCTAT |
| 2732R | TCGAACCATCGGACGTTCAG |
| E1998F | ACGCGTCGACTGGTCACCAAAACATATTTTAATTTATTG (*Sal* I) | Heterologous expression in *E.coli* |
| E1998R | ATAAGAATGCGGCCGCCTCTATCTTCCTTTCTTTTCTTCTTAC (*Not* I) |
| E1998/93R | ATAAGAATGCGGCCGCAGGACTTACTTCATCAAC (*Not* I) |
| E806F | CGGAATTCATGCAAACAATCTATCAGCCAG (*Eco*R I) |
| E806R | CCGCTCGAGATAGGCTTTTTTTACTTCAAC (*Xho* I) |
| E2030F | CGGAATTCATGCAAACAATCTACCAACCTG (*Eco*R I) |
| E2030R | CCGCTCGAGGTATGCTTTGGACAGTGTA (*Xho* I) |
| E805F | CGGAATTCATGAATATTAATCTGGAAAATC (*Eco*R I) |
| E805R | CCGCTCGAGTTTCATAAAGAGTCCGCCGT (*Xho* I) |
| E2859F | GGAATTCCATATGAGAAGAAGAATGATTATC (*Nde* I) |
| E2859R | ACGCGTCGACGTATGAAATAGACTCAAC (*Sal* I) |
| E3263F | ACGCGTCGACTGAAAAAACTACTAGCTATTTTG (*Sal* I) |
| E3263R | CCGCTCGAGGTTCATTCTTCTTTTTT (*Xho* I) |
| E3268F | ACGCGTCGACTGGAAAAAGATCGAACATTAC (*Sal* I) |
| E3268R | CCGCTCGAGCACCTCTGTATGTTTTTG (*Xho* I) |

**Table S2. Gene loci for mono- and disaccharides uptake and utilization**

| **Type of saccharides** | **Locus** | **Gene ID** | **Annotation** |
| --- | --- | --- | --- |
| ribose | Locus 1 | 395 | Ribose operon repressor |
|  |  | 396 | Ribokinase (EC 2.7.1.15) |
|  |  | 397 | Ribose ABC transport system, high affinity permease RbsD (TC 3.A.1.2.1) |
|  |  | 398 | Ribose ABC transport system, ATP-binding protein RbsA (TC 3.A.1.2.1) |
|  |  | 399 | Ribose ABC transport system, permease protein RbsC (TC 3.A.1.2.1) |
|  |  | 400 | Ribose ABC transport system, periplasmic ribose-binding protein RbsB (TC 3.A.1.2.1) |
|  | Locus 2 | 3694 | Ribose ABC transport system, permease protein RbsC (TC 3.A.1.2.1) |
|  |  | 3695 | Ribose ABC transport system, ATP-binding protein RbsA (TC 3.A.1.2.1) |
|  |  | 3696 | putative sugar ABC transporter |
|  | Locus 3 | 2392 | Ribulose-phosphate 3-epimerase (EC 5.1.3.1) |
|  | Locus 4 | 548 | transketolase |
| xylose | Locus 1 | 322 | Xylose isomerase domain protein TIM barrel |
|  | Locus 2 | 3273 | Xylose ABC transporter, substrate-binding component |
|  |  | 3274 | hypothetical protein |
|  |  | 3275 | Xylose ABC transporter, permease component |
|  |  | 3276 | Xylose ABC transporter, permease component |
|  | Locus 3 | 548 | transketolase |
| galactose | Locus 1 | 2982 | Galactose-1-phosphate uridylyltransferase (EC 2.7.7.10) |
|  |  | 2983 | UDP-glucose 4-epimerase (EC 5.1.3.2) |
|  |  | 2984 | Galactokinase (EC 2.7.1.6) |
|  |  | 2985 | Galactose operon repressor, GalR-LacI family of transcriptional regulators |
| mannose | Locus 1 | 3718 | PTS system, mannose-specific IIB component (EC 2.7.1.69) / PTS system, mannose-specific IIC component (EC 2.7.1.69) / PTS system, mannose-specific IIA component (EC 2.7.1.69) |
|  |  | 3719 | hypothetical protein |
|  |  | 3720 | Activator of the mannose operon (transcriptional antiterminator), BglG family |
|  | Locus 2 | 3415 | Phosphomannomutase (EC 5.4.2.8) |
|  | Locus 3 | 426 | Mannose-6-phosphate isomerase (EC 5.3.1.8) |
|  | Locus 4 | 498 | Mannose-6-phosphate isomerase (EC 5.3.1.8) |
|  | Locus 5 | 2079 | Mannose-6-phosphate isomerase (EC 5.3.1.8) |
|  | Locus 6 | 355 | Mannose-1-phosphate guanylyltransferase (GDP) (EC 2.7.7.22) |
|  | Locus 7 | 3932 | Mannose-1-phosphate guanylyltransferase (GDP) (EC 2.7.7.22) |
|  |  | 3933 | GDP-mannose 4,6-dehydratase (EC 4.2.1.47) |
| mannitol | Locus 1 | 421 | PTS system, mannitol-specific IIC component (EC 2.7.1.69) / PTS system, mannitol-specific IIB component (EC 2.7.1.69) / PTS system, mannitol-specific IIA component |
|  |  | 422 | Mannitol operon activator, BglG family |
|  |  | 423 | Mannitol-1-phosphate 5-dehydrogenase (EC 1.1.1.17) |
| glucose | Locus 1 | 2659 | Phosphoenolpyruvate-protein phosphotransferase of PTS system (EC 2.7.3.9) |
|  |  | 2660 | Phosphocarrier protein of PTS system |
|  |  | 2661 | PTS system, glucose-specific IIC component / PTS system, glucose-specific IIB component (EC 2.7.1.69) / PTS system, glucose-specific IIA component |
|  |  | 2662 | Beta-glucoside bgl operon antiterminator, BglG family |
| fructose | Locus 1 | 906 | Transcriptional repressor of the fructose operon, DeoR family |
|  | Locus 2 | 2570 | PTS system, fructose-specific IIA component (EC 2.7.1.69) / PTS system, fructose-specific IIB component (EC 2.7.1.69) / PTS system, fructose-specific IIC component (EC 2.7.1.69) |
|  |  | 2571 | 1-phosphofructokinase (EC 2.7.1.56) |
|  |  | 2572 | Transcriptional repressor of the fructose operon, DeoR family |
|  |  | 2573 | ABC transporter, ATP-binding protein |
|  |  | 2574 | ABC transporter, ATP-binding protein |
|  |  | 2575 | periplasmic component of efflux system |
| maltose | Locus 1 | 104 | Maltose operon transcriptional repressor MalR, LacI family |
|  | Locus 2 | 302 | Maltose/maltodextrin ABC transporter, permease protein MalF |
|  |  | 303 | Maltose/maltodextrin ABC transporter, substrate binding periplasmic protein MalE |
|  |  | 304 | Maltose/maltodextrin ABC transporter, permease protein MalG |
|  | Locus 3 | 2938 | Alpha-glucosidase (EC 3.2.1.20) |
|  |  | 2939 | Maltose operon transcriptional repressor MalR, LacI family |
|  | Locus 4 | 3010 | Alpha-glucosidase (EC 3.2.1.20) |
| lactose | Locus 1 | 105 | Beta-galactosidase (EC 3.2.1.23) |
|  | Locus 2 | 3434 | Alpha-galactosidase (EC 3.2.1.22) |
|  |  | 3435 | Beta-galactosidase (EC 3.2.1.23) |
|  | Locus 3 | 3438 | lactose transport system (lactose-binding protein) |
| trehalose | Locus 1 | 22 | Trehalose operon transcriptional repressor |
|  |  | 23 | Trehalose-6-phosphate hydrolase (EC 3.2.1.93) |
|  |  | 24 | PTS system, trehalose-specific IIB component (EC 2.7.1.69) / PTS system, trehalose-specific IIC component (EC 2.7.1.69) |
|  | Locus 2 | 3646 | Trehalose-6-phosphate hydrolase (EC 3.2.1.93) |
|  |  | 3647 | PTS system, trehalose-specific IIB component (EC 2.7.1.69) / PTS system, trehalose-specific IIC component (EC 2.7.1.69) |
|  | Locus 3 | 4266 | Trehalose operon transcriptional repressor |
|  |  | 4267 | Trehalose-6-phosphate hydrolase (EC 3.2.1.93) |

**Table S3. Classification of carbohydrate-active enzymes (CAZy), including GHs, GTs, PLs, CEs, and CBMs**

| **Gene ID** | **CAZy family** | **Annotation** |
| --- | --- | --- |
| **Glycoside Hydrolases (GHs)** | | |
| 23 | GH13 | Trehalose-6-phosphate hydrolase |
| 88 | GH14 | Beta-amylase (EC 3.2.1.2) |
| 105 | GH1 | Beta-galactosidase (EC 3.2.1.23) |
| 194 | GH31 | Maltodextrin glucosidase (EC 3.2.1.20) |
| 358 | GH73 | Bifunctional autolysin Atl / N-acetylmuramoyl-L-alanine amidase (EC 3.5.1.28)/ endo-beta-N-acetylglucosaminidase (EC 3.2.1.96) |
| 823 | GH13 | 1,4-alpha-glucan (glycogen) branching enzyme, GH-13-type (EC 2.4.1.18) |
| 919 | GH13 | Glycogen debranching enzyme (EC 3.2.1.-) / Pullulanase (EC 3.2.1.41) |
| 2206 | GH109 | Myo-inositol 2-dehydrogenase (EC 1.1.1.18) |
| 2227 | GH109 | predicted dehydrogenase |
| 2228 | GH109 | Gluconokinase (EC 2.7.1.12) / oxidoreductase domain |
| 2732 | GH13 | Pullulanase (EC 3.2.1.41) |
| 2859 | GH16 | Endo-beta-1,3-1,4 glucanase (Licheninase) (EC 3.2.1.73) |
| 2938 | GH13 | Alpha-glucosidase (EC 3.2.1.20) |
| 3010 | GH31 | Alpha-glucosidase (EC 3.2.1.20) |
| 3015 | GH109 | Myo-inositol 2-dehydrogenase (EC 1.1.1.18) |
| 3123 | GH23 | Lytic transglycosylase |
| 3188 | GH13 | Periplasmic alpha-amylase (EC 3.2.1.1) |
| 3263 | GH16 | Beta-glucanase precursor (EC 3.2.1.73) |
| 3265 | GH30 | O-Glycosyl hydrolase family 30 |
| 3267 | GH1 | Beta-glucosidase (EC 3.2.1.21) |
| 3268 | GH16 | Beta-glucanase precursor (EC 3.2.1.73) |
| 3378 | GH109 | Possible oxidoreductase |
| 3395 | GH74 | VPS10, VPS10 domain |
| 3434 | GH36 | Alpha-galactosidase (EC 3.2.1.22) |
| 3435 | GH2 | Beta-galactosidase (EC 3.2.1.23) |
| 3547 | GH109 | NADH-dependent dehydrogenase |
| 3548 | GH109 | Myo-inositol 2-dehydrogenase (EC 1.1.1.18) |
| 3635 | GH25 | lysozyme, putative |
| 3646 | GH13 | Trehalose-6-phosphate hydrolase (EC 3.2.1.93) |
| 3712 | GH105 | FIG01249104: hypothetical protein |
| 4109 | GH18 | Spore cortex-lytic enzyme, N-acetylglucosaminidase SleL (EC 3.2.1.-) |
| 4267 | GH13 | Trehalose-6-phosphate hydrolase (EC 3.2.1.93) |
| **Glycosyl Transferases (GTs)** | | |
| 82 | GT51 | Multimodular transpeptidase-transglycosylase(EC 2.4.1.129) (EC 3.4.-.-) |
| 228 | GT4 | Lipid carrier : UDP-N-acetylgalactosaminyltransferase (EC 2.4.1.-) / Alpha-1,3-N-acetylgalactosamine transferase PglA (EC 2.4.1.-); Putative glycosyltransferase |
| 232 | GT2 | glycosyltransferase |
| 234 | GT4 | Alpha-1,4-N-acetylgalactosamine transferase PglJ (EC 2.4.1.-) |
| 235 | GT2 | Glycosyltransferase (EC 2.4.1.-) |
| 356 | GT94 | putative glycosyl transferase |
| 359 | GT4 | Glycosyltransferase |
| 362 | GT2 | Glycosyltransferase |
| 364 | GT2 | Glycosyl transferase, group 2 family protein |
| 721 | GT51 | Multimodular transpeptidase-transglycosylase (EC 2.4.1.129) (EC 3.4.-.-) |
| 743 | GT2 | Glycosyl transferase, group 2 family protein |
| 826 | GT5 | Glycogen synthase, ADP-glucose transglucosylase (EC 2.4.1.21) |
| 827 | GT35 | Glycogen phosphorylase (EC 2.4.1.1) |
| 878 | GT4 | Glycosyl transferase, group 1 family (EC 2.4.1.-) |
| 946 | GT51 | Multimodular transpeptidase-transglycosylase (EC 2.4.1.129) (EC 3.4.-.-) |
| 1316 | GT4 | Glycosyltransferase (EC 2.4.1.-) |
| 1593 | GT4 | Glycosyl transferase, group 1 family protein |
| 1606 | GT51 | Multimodular transpeptidase-transglycosylase (EC 2.4.1.129) (EC 3.4.-.-) / Penicillin-binding protein 1A/1B (PBP1) |
| 1661 | GT28 | diglucosyldiacylglycerol synthase (LTA membrane anchor synthesis) |
| 2450 | GT28 | UDP-N-acetylglucosamine--N-acetylmuramyl-(pentapeptide) pyrophosphoryl-undecaprenol N-acetylglucosamine transferase (EC 2.4.1.227) |
| 2672 | GT2 | Glycosyl transferase, family 2 |
| 2788 | GT2 | Glycosyl transferase, family 2 |
| 2789 | GT2 | Glycosyltransferase |
| 2877 | GT2 | Cytochrome c-type biogenesis protein DsbD, protein-disulfide reductase (EC 1.8.1.8) |
| 3261 | GT51 | Multimodular transpeptidase-transglycosylase (EC 2.4.1.129) (EC 3.4.-.-) |
| 3302 | GT46×2 | hypothetical protein |
| 3304 | GT2 | probable glycosyl transferase |
| 3574 | GT2 | 4,4'-diaponeurosporenoate glycosyltransferase (EC 2.4.1.-) |
| 3681 | GT21 | Cell wall-binding protein |
| 3938 | GT2 | Glycosyl transferase, group 2 family protein |
| 3939 | GT4×2 | Glycosyl transferase family 1 protein-like |
| **Polysaccharide Lyases (PLs)** | | |
| 806 | PL15 | oligo alginate lyase |
| 1998 | PL17 | F5/8 type C domain protein |
| 2030 | PL15 | oligo alginate lyase |
| **Carbohydrate Esterases (CEs)** | | |
| 130 | CE1 | putative alpha-dextrin endo-1,6-alpha-glucosidase |
| 485 | CE1 | Carboxylesterase (EC 3.1.1.1) |
| 533 | CE1 | Lysophospholipase (EC 3.1.1.5); Monoglyceride lipase (EC 3.1.1.23); putative |
| 729 | CE1 | Hydrolase, alpha/beta fold family |
| 835 | CE1 | 2-succinyl-6-hydroxy-2,4-cyclohexadiene-1-carboxylate synthase (EC 4.2.99.20) |
| 862 | CE10 | Dipeptidyl aminopeptidases/acylaminoacyl-peptidases |
| 881 | CE1 | Lysophospholipase (EC 3.1.1.5); Monoglyceride lipase (EC 3.1.1.23); putative |
| 1078 | CE1 | hypothetical protein |
| 1475 | CE1; CE7 | YqkD |
| 1592 | CE14 | Lmbe-related protein |
| 1686 | CE3 | Lipase/Acylhydrolase with GDSL-like motif |
| 1693 | CE1 | Alpha/beta hydrolase fold |
| 1735 | CE14 | FIG013761: LmbE family protein |
| 1774 | CE1 | hydrolase |
| 1824 | CE1 | Carboxylesterase (EC 3.1.1.1) |
| 1927 | CE1 | Hydrolase of unknown specificity RsbQ, part of a novel [RsbQ - PAS domain] bacterial sensing module |
| 1969 | CE1 | FIG01231988: hypothetical protein |
| 2298 | CE4 | FIG007013: polysaccharide deacetylase, putative |
| 2923 | CE1 | Menaquinone biosynthesis related protein MenX |
| 3085 | CE1 | Putative acetyl esterase YjcH (EC 3.1.1.-) |
| 3168 | CE1 | Putative hydrolase of the alpha/beta superfamily |
| 3236 | CE9 | N-acetylglucosamine-6-phosphate deacetylase (EC 3.5.1.25) |
| 3480 | CE1×2 | putative esterase |
| 3553 | CE1 | 3-Oxoadipate enol-lactonase, alpha/beta hydrolase fold family [EC:3.1.1.24] |
| 3622 | CE4 | Polysaccharide deacetylase |
| 3737 | CE1 | Poly(3-hydroxybutyrate) depolymerase |
| 3745 | CE7 | Hydrolases of the alpha/beta superfamily |
| 3928 | CE1; CE7 | putative esterase/lipase |
| 3957 | CE4 | polysaccharide deacetylase, putative |
| **Carbohydrate-Binding Modules (CBMs)** | | |
| 88 | CBM20 | Beta-amylase (EC 3.2.1.2) |
| 559 | CBM50×7 | Membrane-bound lytic murein transglycosylase D precursor (EC 3.2.1.-) |
| 788 | CBM50×2 | Cell wall-binding protein |
| 823 | CBM48 | 1,4-alpha-glucan (glycogen) branching enzyme, GH-13-type (EC 2.4.1.18) |
| 919 | CBM48 | Glycogen debranching enzyme (EC 3.2.1.-) / Pullulanase (EC 3.2.1.41) |
| 1066 | CBM51 | hypothetical protein |
| 1115 | CBM50 | Stage VI sporulation protein D |
| 1163 | CBM50 | Gamma-aminobutyrate:alpha-ketoglutarate aminotransferase (EC 2.6.1.19) |
| 1524 | CBM50×2 | peptidase, M23/M37 family |
| 1530 | CBM50 | hypothetical protein |
| 1740 | CBM50×3 | Membrane-bound lytic murein transglycosylase D precursor (EC 3.2.1.-) |
| 1771 | CBM50 | Transporter |
| 1998 | CBM32 | F5/8 type C domain protein |
| 2004 | CBM32 | Polyguluronate lyase precursor (EC 4.2.2.11) |
| 2092 | CBM50 | Spore cortex-lytic enzyme, lytic transglycosylase SleB |
| 2224 | CBM50 | ABC transport protein, sugar-binding component yneA |
| 2622 | CBM50 | Protein erfK/srfK precursor |
| 2684 | CBM50 | Spore cortex-lytic enzyme |
| 2732 | CBM48×3; CBM20; CBM41 | Pullulanase (EC 3.2.1.41) |
| 2987 | CBM40 | Uncharacterized iron-regulated membrane protein; Iron-uptake factor PiuB |
| 3263 | CBM4×4 | Beta-glucanase precursor (EC 3.2.1.73) |
| 4109 | CBM50×2 | Spore cortex-lytic enzyme, N-acetylglucosaminidase SleL (EC 3.2.1.-) |
| 4153 | CBM50 | Membrane proteins related to metalloendopeptidases |

**Table S4. Up-regulated genes predicted by RNA-seq based transcriptomic analysis**

| **Gene ID** | **log_2_FoldChange** | **adjusted P value** | **Annotation** |
| --- | --- | --- | --- |
| 394 | 3.5764 | 0.040181 | Ribosomal subunit interface protein |
| 395 | 3.3696 | 0.033293 | Ribose operon repressor |
| 397 | 4.6189 | 0.018597 | Ribose ABC transport system, high affinity permease RbsD |
| 398 | 3.6185 | 0.018597 | Ribose ABC transport system, ATP-binding protein RbsA |
| 399 | 3.6657 | 0.01994 | Ribose ABC transport system, permease protein RbsC |
| 503 | 5.27 | 0.000079358 | 2-dehydro-3-deoxygluconate kinase |
| 504 | 6.1632 | 0.0000074226 | 2-dehydro-3-deoxyphosphogluconate aldolase |
| 534 | 3.9374 | 0.0079075 | cheX protein |
| 571 | 4.1757 | 0.0050804 | NADH dehydrogenase |
| 646 | 3.3629 | 0.01994 | Homoserine kinase |
| 800 | 6.1963 | 0.049561 | ABC-type polysaccharide transport system, permease component |
| 801 | 6.929 | 0.00000073093 | putative transport system integral membrane protein |
| 802 | 4.2361 | 0.0031858 | ABC transporter, substrate-binding protein |
| 804 | 3.3014 | 0.028459 | two-component system sensor kinase |
| 805 | 5.988 | 0.0000089097 | 3-oxoacyl-[acyl-carrier protein] reductase |
| 806 | 6.2298 | 0.0000059362 | oligo alginate lyase |
| 807 | 5.9675 | 0.000015607 | pectin degradation protein |
| 1226 | 4.7043 | 0.0054627 | hypothetical protein |
| 1791 | 4.2461 | 0.0019398 | Glutamate synthase [NADPH] large chain |
| 1792 | 3.7616 | 0.010904 | Glutamate synthase [NADPH] small chain |
| 1998 | 4.188 | 0.0031858 | F5/8 type C domain protein |
| 2414 | 3.5144 | 0.026891 | Sulfate adenylyltransferase, dissimilatory-type |
| 2415 | 3.5516 | 0.022979 | Sulfate permease, Pit-type |
| 2416 | 4.8443 | 0.00080596 | Phosphoadenylyl-sulfate reductase [thioredoxin] |
| 2420 | 4.7522 | 0.0018486 | Dihydroorotate dehydrogenase, catalytic subunit |
| 2941 | 4.5147 | 0.0033331 | Multiple sugar ABC transporter, membrane-spanning permease protein MsmG |
| 2942 | 3.9512 | 0.0080725 | Multiple sugar ABC transporter, membrane-spanning permease protein MsmF |
| 3341 | 4.5734 | 0.001658 | Multiple sugar ABC transporter, ATP-binding protein |
| 3365 | 3.1944 | 0.037128 | Sulfate permease |
| 3403 | 4.1952 | 0.0035535 | Flavohemoprotein (Hemoglobin-like protein) (Flavohemoglobin) (Nitric oxide dioxygenase) |
| 3531 | 3.3013 | 0.027144 | Metallo-dependent hydrolase, subgroup C |
| 3564 | 4.7681 | 0.0018743 | Phage infection protein |
| 3565 | 3.2463 | 0.040708 | Transcriptional regulator, TetR family |
| 3646 | 3.269 | 0.033958 | Trehalose-6-phosphate hydrolase |

**Table S5. Genes involved in glycogen synthesis of *B. weihaiensis* Alg07**

| **Gene ID** | **Annotation** |
| --- | --- |
| 823 | 1,4-alpha-glucan (glycogen) branching enzyme, GH-13-type (EC 2.4.1.18) |
| 824 | Glucose-1-phosphate adenylyltransferase (EC 2.7.7.27) |
| 825 | Glycogen biosynthesis protein GlgD, glucose-1-phosphate adenylyltransferase family |
| 826 | Glycogen synthase, ADP-glucose transglucosylase (EC 2.4.1.21) |
| 827 | Glycogen phosphorylase (EC 2.4.1.1) |

**Fig. S1. The plasmid of strain Alg07**

**
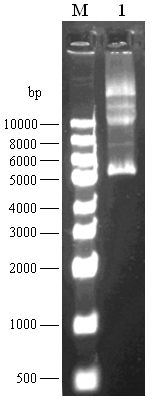
**

**Fig. S2. Heat map of 104 genes whose mRNA level significantly changed.**

**
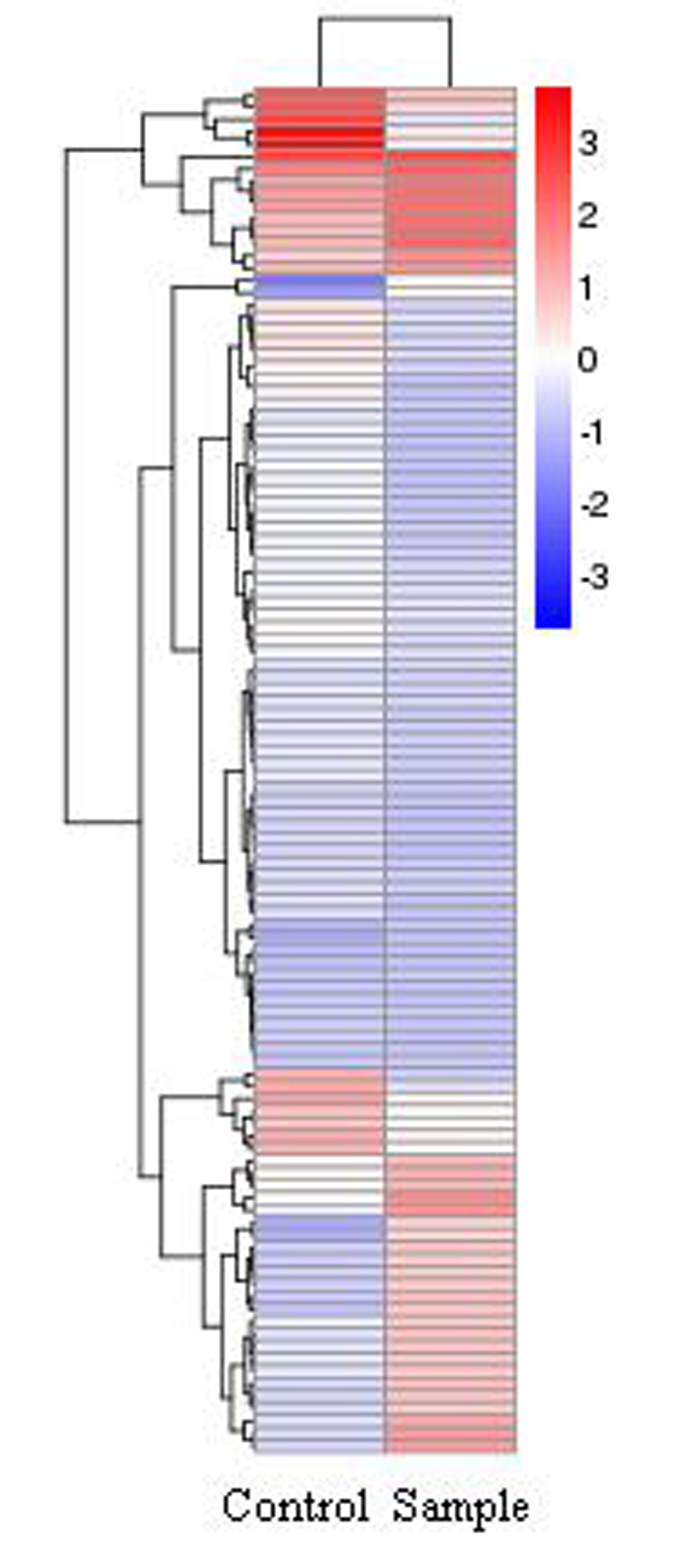
**

**Fig. S3. SDS-PAGE analysis of the heterologous expression of key genes involved in alginate (A) and laminarin (B) degradation.** Lane M, protein marker; lane 1 & 7, the extract of *E. coli* cells containing pET-21a; lane 2, the extract of *E. coli* cells containing pET-Bw1998; lane 3, the extract of *E. coli* cells containing pET-Bw1998/93; lane 4, the extract of *E. coli* cells containing pET-Bw806; lane 5, the extract of *E. coli* cells containing pET-Bw2030; lane 6, the extract of *E. coli* cells containing pET-Bw805; lane 8, the extract of *E. coli* cells containing pET-Bw2859; lane 9, the extract of *E. coli* cells containing pET-Bw3263; lane 10, the extract of *E. coli* cells containing pET-Bw3268.


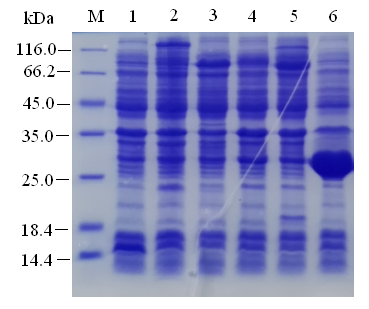


(A)


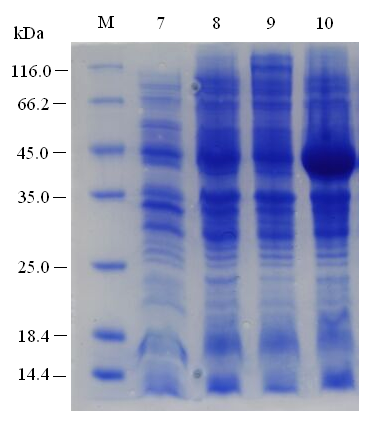


(B)

**Fig. S4. The properties of the recombinant alginate lyase (Bw1998) of *B. weihaiensis* Alg07.** (A) effect of temperature on the activity of alginate lyase; (B) effect of pH on the activity of alginate lyase; (C) effect of metal ion on the activity of alginate lyase; (D) the activity of alginate lyase towards polyM (blue) and polyG (red).


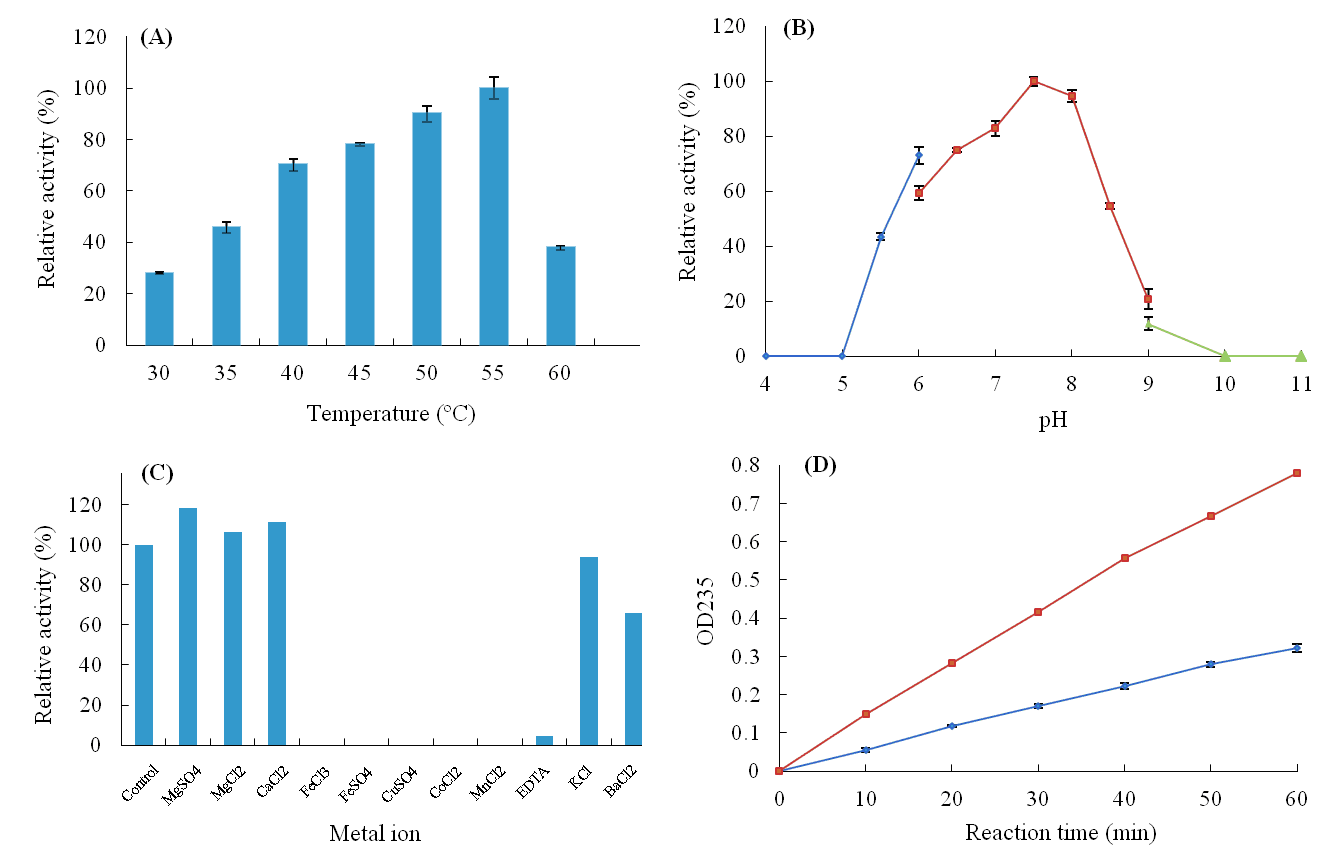


**Fig. S5. Comparison of amino-acid sequences between the DEH reductase (Bw805) of *B. weihaiensis* Alg07 and other reported DEH reductases.** SpDEHR, the DEH reductase from *Sphingomonas* sp. A1; FlDEHR, the DEH reductase from *Flavobacterium* sp. UMI-01. Pentacles represent the residues configuring catalytic tetrad. Open circles show the residues conserved in the Rossmann fold cofactor-binding motif Thr-Gly-X-X-X-Gly-X-Gly.


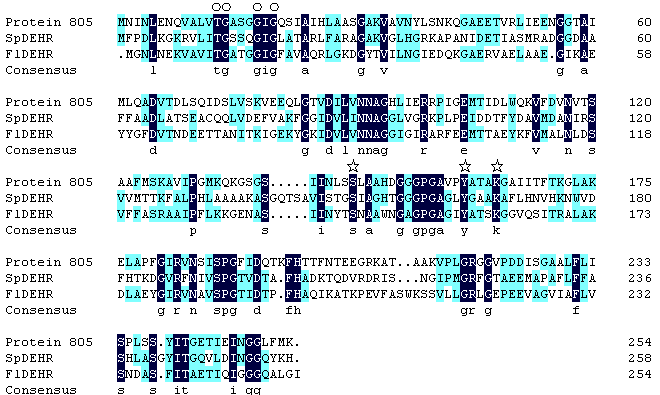

Supplement: Supplementary Information [file srep38248-s1.doc]
